# Glucocorticosteroids Differentially Regulate MMP-9 and Neutrophil Elastase in COPD

**DOI:** 10.1371/journal.pone.0033277

**Published:** 2012-03-07

**Authors:** Ross Vlahos, Peter A. B. Wark, Gary P. Anderson, Steven Bozinovski

**Affiliations:** 1 Department of Pharmacology, The University of Melbourne, Victoria, Australia; 2 Respiratory Medicine HMRI, John Hunter Hospital, Newcastle, NSW, Australia; University of Tübingen, Germany

## Abstract

**Background:**

Chronic Obstructive Pulmonary Disease (COPD) is currently the fifth leading cause of death worldwide. Neutrophilic inflammation is prominent, worsened during infective exacerbations and is refractory to glucocorticosteroids (GCs). Deregulated neutrophilic inflammation can cause excessive matrix degradation through proteinase release. Gelatinase and azurophilic granules within neutrophils are a major source of matrix metalloproteinase (MMP)-9 and neutrophil elastase (NE), respectively, which are elevated in COPD.

**Methods:**

Secreted MMP-9 and NE activity in BALF were stratified according to GOLD severity stages. The regulation of secreted NE and MMP-9 in isolated blood neutrophils was investigated using a pharmacological approach. *In vivo* release of MMP-9 and NE in mice exposed to cigarette smoke (CS) and/or the TLR agonist lipopolysaccharide (LPS) in the presence of dexamethasone (Dex) was investigated.

**Results:**

Neutrophil activation as assessed by NE release was increased in severe COPD (36-fold, GOLD II vs. IV). MMP-9 levels (8-fold) and activity (21-fold) were also elevated in severe COPD, and this activity was strongly associated with BALF neutrophils (r = 0.92, p<0.001), but not macrophages (r = 0.48, p = 0.13). *In vitro*, release of NE and MMP-9 from fMLP stimulated blood neutrophils was insensitive to Dex and attenuated by the PI3K inhibitor, wortmannin. *In vivo*, GC resistant neutrophil activation (NE release) was only seen in mice exposed to CS and LPS. In addition, GC refractory MMP-9 expression was only associated with neutrophil activation.

**Conclusions:**

As neutrophils become activated with increasing COPD severity, they become an important source of NE and MMP-9 activity, which secrete proteinases independently of TIMPs. Furthermore, as NE and MMP-9 release was resistant to GC, targeting of the PI3K pathway may offer an alternative pathway to combating this proteinase imbalance in severe COPD.

## Introduction

Proteinases generated within the lung environment regulate many physiological processes during infection, inflammation and subsequent tissue repair. Innate immune cells including macrophages and neutrophils are a major source of matrix metalloproteinase-9 (MMP-9) and neutrophil elastase (NE), which are central to these processes. In the normal setting, anti-proteinases such as α1-antitrypsin (α1-AT), secretory leukoprotease inhibitor (SLPI) and tissue inhibitor of metalloproteinases (TIMPs) are in excess and provide an anti-proteinase screen to prevent deleterious effects [1]. In lung diseases such as chronic obstructive pulmonary disease (COPD) there is an imbalance leading to excessive proteinase activity that can cause host tissue damage [1]. Activated neutrophils release serine proteinases including NE, Proteinase-3 (PR-3) and MMPs including MMP-9 and MMP-8, which have been shown to be elevated in COPD (reviewed in [2]). COPD is primarily caused by cigarette smoke exposure and is already the fifth leading cause of death worldwide [3]. As the disease progresses, increased susceptibility to respiratory virus and/or bacteria promote acute exacerbations (AECOPD) that further amplifies inflammation [4], [5]. Glucocorticosteroids (GCs) are a mainstay for current AECOPD management, although their efficacy in COPD is suboptimal as neutrophilic inflammation persists following GC treatment [6].

Elevated MMP-9 levels in COPD are related to sputum neutrophil numbers to suggest that this cell type is a major source [7], [8]. Neutrophils contain tertiary gelatinase granules formed at later stages of myelopoiesis that act as a major reservoir for the rapid exocytosis of MMP-9 [9]. This can result in increased local proteolytic activity because unlike other mononuclear leukocytes, this cell type does not express the inhibitor, TIMP1 [10]. MMP-9 degrades collagen, elastin and gelatin and its levels inversely correlate with airflow obstruction [7]. Extracellular matrix derived N-acetyl Pro-Gly-Pro (Ac-PGP) also augments MMP-9 release from neutrophils [11]. NE is abundant in primary azurophil granules formed during the early stages of myelopoiesis and is secreted during neutrophil degranulation [9]. Free NE activity may also accumulate in airways from necrotic neutrophils that release their intracellular content. NE activity is elevated in COPD and the degree of NE localised to lung elastic fibers correlates with the degree of emphysema [12]. NE can degrade extracellular matrix components including elastin, collagens I-IV and fibrinogen and the genetic deficiency in α1-AT is associated with early onset pan lobular emphysema [13]. Serine proteinases such as NE are considered to be at the apex of the proteinase hierarchy in airways as they have the ability to activate MMPs including MMP-9 via cleavage of pro-MMP-9 into active-MMP-9 [14]. Furthermore, NE preferentially degrades TIMP1 that is bound to MMP-9, thereby liberating active MMP-9 [15]. NE can also activate the inflammatory NFκB pathway via a Toll-Like Receptor-4 (TLR-4) dependent manner and promote an acute inflammatory response [16]. The promoter of MMP-9 also contains an NFκB binding site that promotes its expression [17].

Here, we have directly measured secreted MMP-9 and NE in BAL–fluid (BALF) from a COPD cohort stratified according to GOLD stages and have shown that NE and MMP-9 activity increase with disease severity. In addition, neutrophils were strongly related to both proteinases in severe COPD. We hypothesised that neutrophils are an important source of NE and MMP-9 that is insensitive to GCs. To examine this, we have assessed their release from isolated blood neutrophils in the presence of pharmacological inhibitors and in an *in vivo* inflammatory model that combines CS exposure and acute inflammation (LPS).

## Results

### Airway neutrophils release MMP-9 and NE in COPD

**Figure 1:**
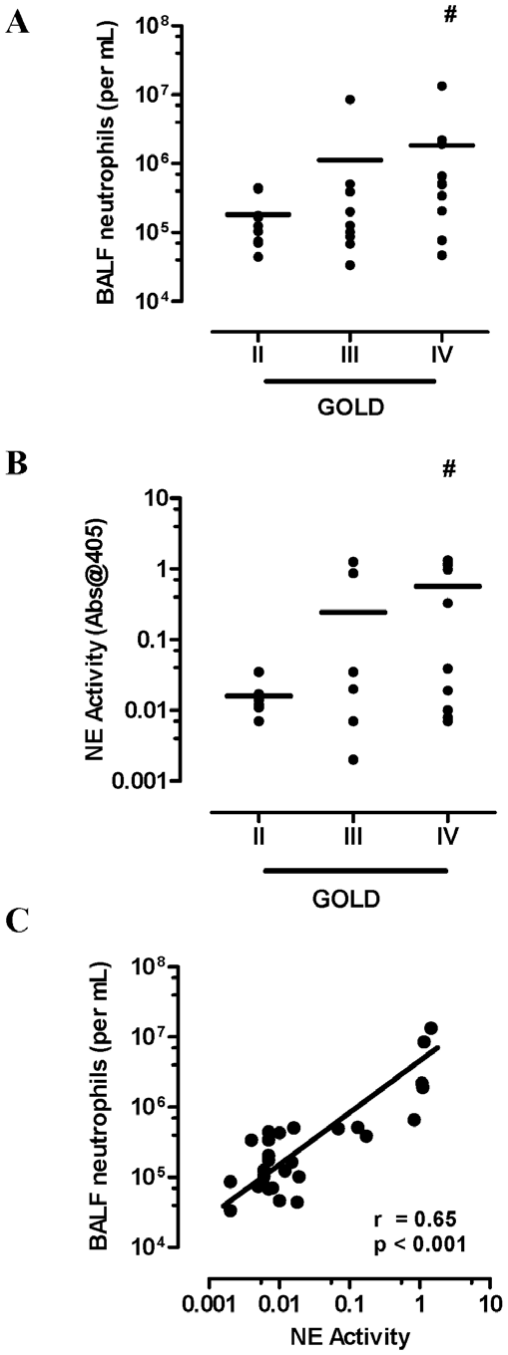
Neutrophilic airway inflammation increases with disease severity. (A) BALF neutrophils were higher in very severe COPD (GOLD IV) compared to participants with moderate COPD (GOLD II). (B) NE activity stratified across the GOLD stages demonstrates increased NE activity in GOLD IV. (C) Neutrophils were positively related to NE activity (Spearman r = 0.65, *p<0.001*) in BALF. Individual data points are shown with line representing mean values. *# p<0.05* versus GOLD II.

COPD severity was classified as moderate (stage II, n = 9 (31%)), severe (stage III, n = 9 (31%)), and very severe (stage IV, n = 11 (38%)) according to GOLD criteria as previously described [18]. Inhaled corticosteroid (ICS) daily usage increased across the severity grades as summarised in Table 1. Neutrophil BALF numbers increased with disease severity (GOLD II; mean 0.18 (range 0.04–0.45), GOLD III; 1.12 (0.03–8.5) and GOLD IV; 1.83 (0.05–13.4) neutrophils (×10^6^ per mL) with a significant increase observed in GOLD IV vs. GOLD II (p<0.05, Figure 1A). NE activity also increased with disease severity (GOLD II; mean 0.016 (range 0.007–0.04), GOLD III; 0.25 (0.002–1.25) and GOLD IV; 0.57 (.007–1.31) with a significant increase observed in GOLD IV vs. GOLD II (*p<0.05*, Figure 1B). There was a positive correlation between neutrophil BALF numbers and NE activity (Figure 1C; r = 0.65, *p<0.001*).

**Table 1:** COPD patient characteristics.

GOLD Criteria	n	M∶F	Age (range)	Pack Years (range)	% using ICS	#Total daily ICS dose (range)
II	9	5∶4	64 (56–72)	40 (20–70)	3/9 (33%)	311 (0–1000)
III	9	6∶3	70 (60–75)	35 (20–150)	5/9 (56%)	566 (0–1000)
IV	11	7∶4	74 (69–80)	45 (20–116)	10/11 (91%)	1070 (0–1600)

# Total daily ICS dose (beclamethasone equivalents), based on the assumption that 1 µg.

beclomethasone = 1 µg Budesonide = 0.5 µg Fluticasone.

### Increased MMP-9 is associated with increased neutrophilic inflammation in severe COPD

**Figure 2:**
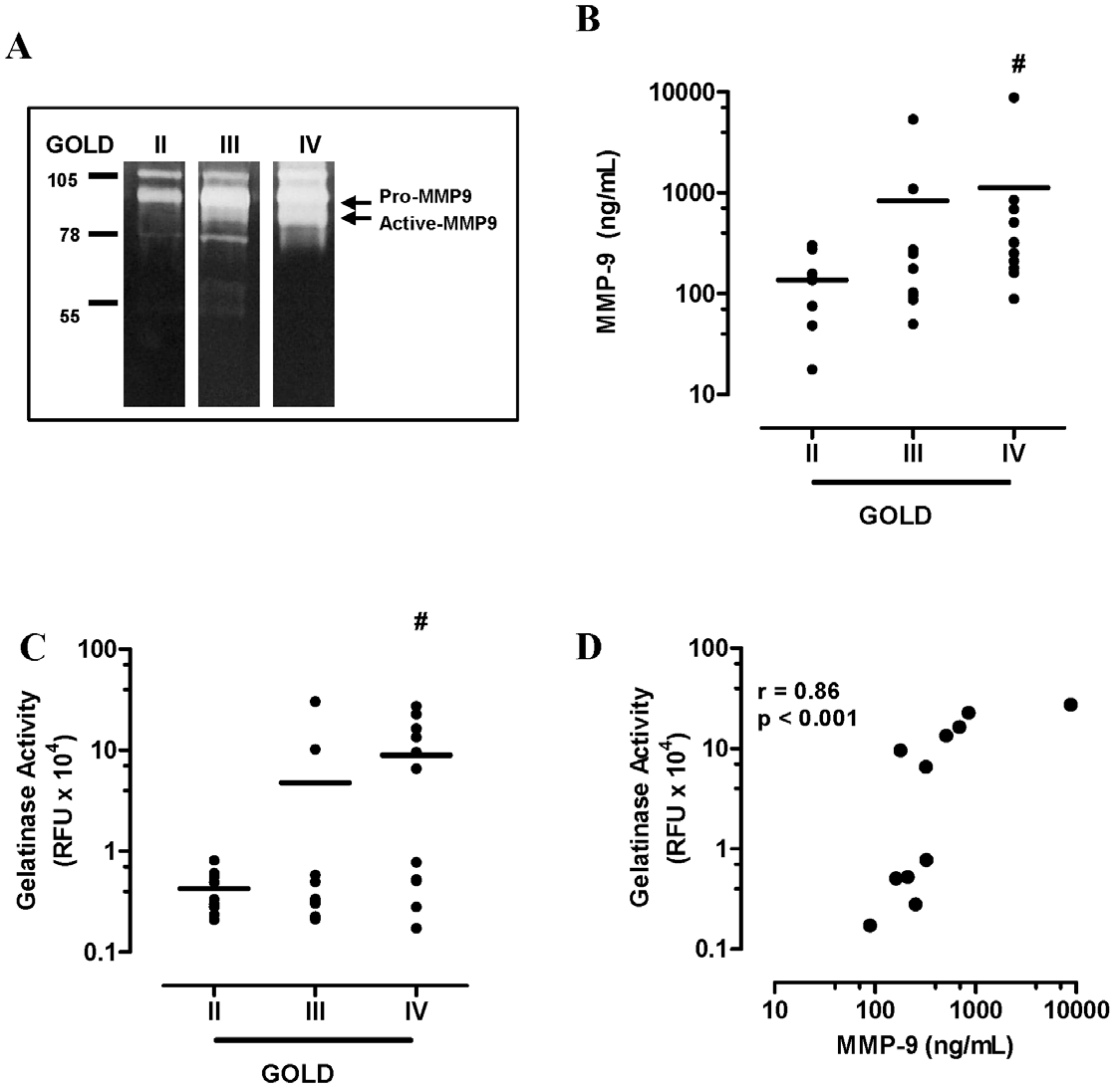
MMP-9 expression and activity increased in severe COPD. (A) Gelatin zymography (representative bands from each GOLD stage) identify major bands of activity correlating to the molecular weight of pro (92 kDa) and active (82 kDa) MMP-9. (B) MMP-9 expression and (C) activity increased with disease severity. (D) MMP-9 expression and gelatinase activity in GOLD IV BALF were strongly related (Spearman r = 0.86, *p<0.001*). Individual data points are shown with line representing mean values. *# p<0.05* versus GOLD II.

**Figure 3:**
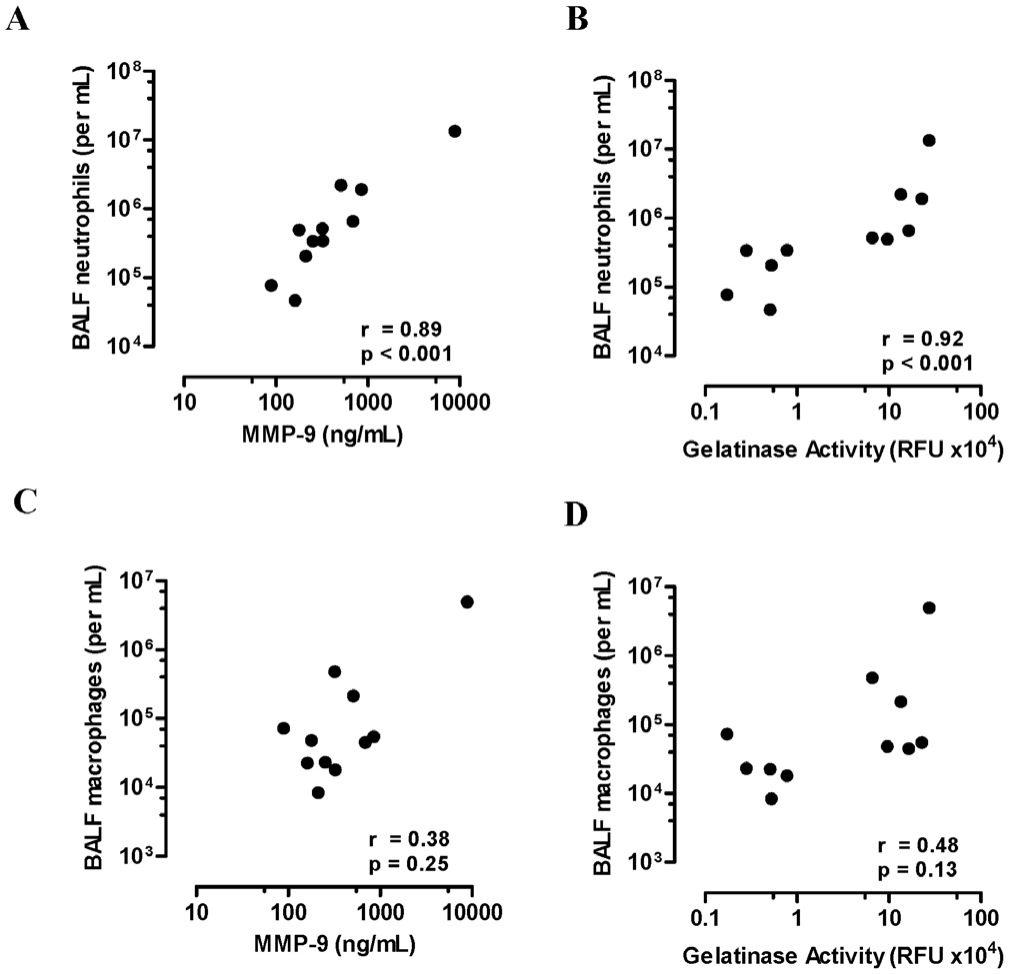
MMP-9 is associated with neutrophilic inflammation in COPD. (A) MMP-9 expression was positively associated with BALF neutrophils in GOLD IV COPD (Spearman r = 0.0.89, *p<0.001*). (B) MMP-9 activity was also positively associated with BALF neutrophils in GOLD IV COPD (Spearman r = 0.0.92, *p<0.001*), whereas no association was observed with BALF macrophages (C & D).

Gelatin zymography of COPD BALF identified major bands of activity within the 90 kDa range, which is consistent with the molecular weight of pro (92 kDa) and active (82 kDa) MMP-9 (Figure 2A). Total MMP-9 (pro and active forms) in BALF was next measured by quantitative ELISA. The expression of MMP-9 increased with COPD severity (GOLD II; mean 137 (range 17.7–301), GOLD III; 833 (50–5360) and GOLD IV; 1127 (88.6–8820) ng/mL with a significant increase observed in GOLD IV vs. GOLD II (*p<0.05*, Figure 2B). Active MMP-9 quantified using flourescein conjugated gelatin as a substrate also showed an increase in MMP-9 activity with COPD severity (GOLD II; mean 0.43 (range 0.21–0.81), GOLD III; 4.78 (0.21–30.4) and GOLD IV; 8.96 (0.17–27.4)×10^4^ RFU, with a significant increase observed in GOLD IV vs. GOLD II (*p<0.05*, Figure 2C). There was a strong correlation between MMP-9 expression and gelatinase activity, confirming MMP-9 as the major gelatinase enzyme in severe COPD BALF (Figure 2D; r = 00.86, *p<0.001*). There was also a strong association between BALF neutrophils and MMP-9 expression (Figure 3A, r = 0.89, *p<0.001*) and activity (Figure 3B, r = 0.92, *p<0.001*) in GOLD IV COPD. BALF macrophages and MMP-9 expression (Figure 3C, r = 0.38, *p = 0.25*) and activity (Figure 3D, r = 0.48, *p = 0.13*) were not significantly associated in severe COPD.

### Stimulated blood neutrophils rapidly release MMP-9 and NE in a GC refractory manner

**Figure 4:**
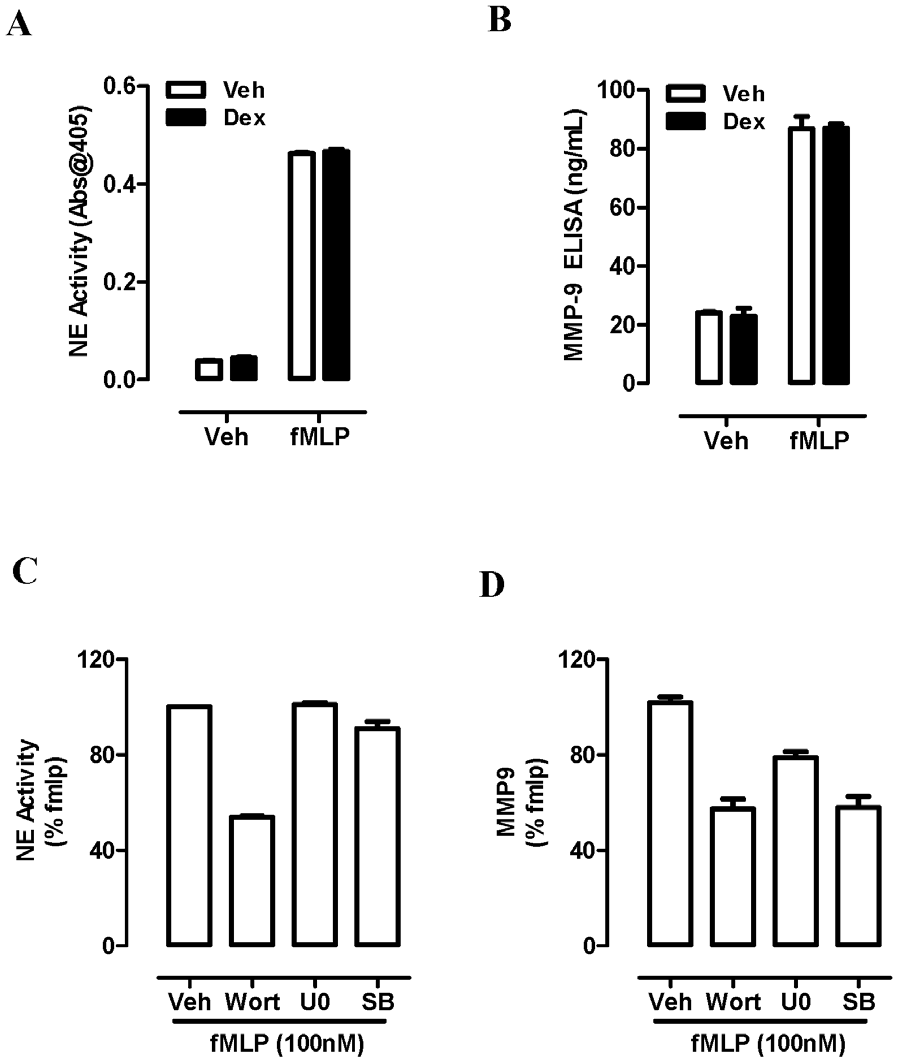
Neutrophils release NE and MMP-9 in a GC resistant manner. Isolated blood neutrophils from healthy volunteers were pretreated with Vehicle (Veh, 0.01% DMSO) or Dex (100 nM) for 15 min prior to fMLP stimulation (100 nM, 20 min at 37C) and (A) NE activity and (B) MMP-9 release was determined. Isolated neutrophils were also pretreated with wortmannin (Wort, 100 nM), U0126 (U0, 1 µM) and SB203580 (SB, 1 µM) prior to fMLP stimulation and cell free supernatants were retained for assessment of (C) NE activity and (D) MMP-9 expression as a percentage of the fMLP positive control. Data is presented as the mean±SEM of the 3 replicates from an individual donor and is representative of 3 separate experiments.

Isolated blood neutrophils stimulated with fMLP rapidly release (20 min) NE (Figure 4A) and MMP-9 (Figure 4B) in a Dex resistant manner. Pretreatment for 15 min prior to fMLP stimulation with the PI3K inhibitor, wortmannin, blocked release of NE and MMP-9 by ∼50% (Figure 4C & D), whereas the MAPK inhibitors (U0126; Erk pathway and SB203580; p38 pathway) were only effective in reducing MMP-9 release by 22% and 42% respectively (Figures 4C & D).

### CS promotes GC resistant airway inflammation

**Figure 5:**
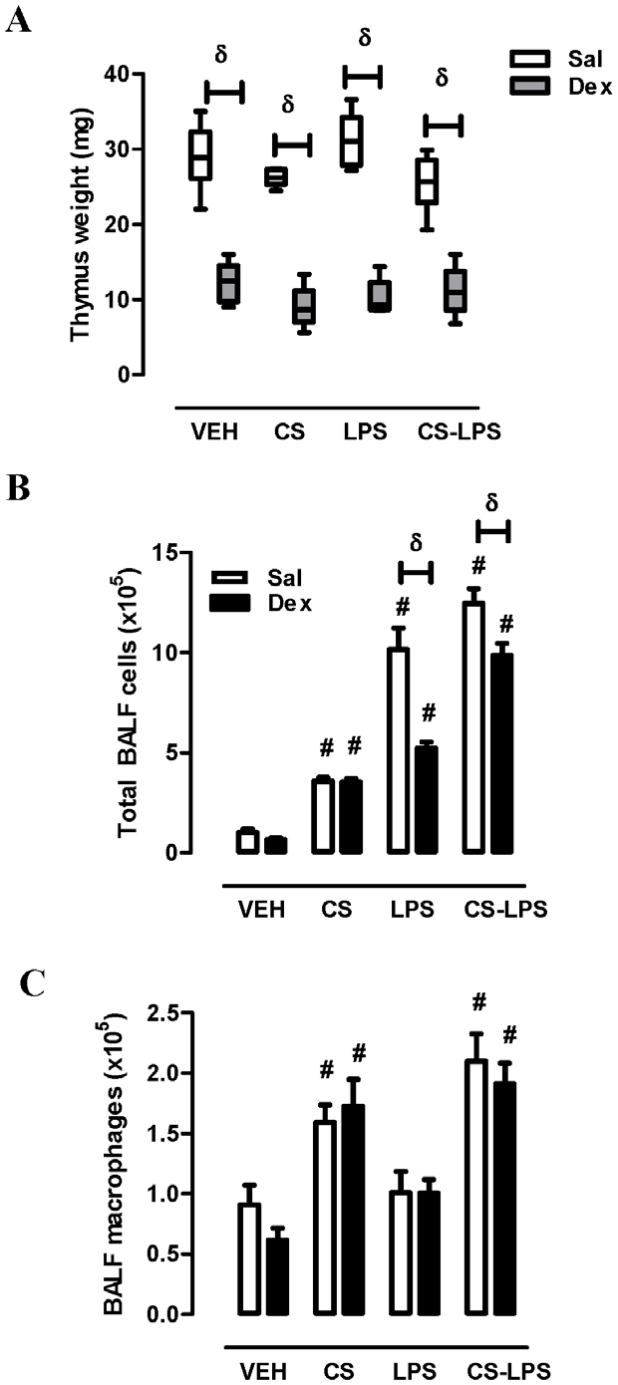
CS exposure reduces the efficacy of Dex to suppress acute airway inflammation. Mice were treated with Saline (open bar) or Dex (closed bar, 1 mg/kg i.p) once daily over 4 days for 2 hours prior to CS exposure as detailed in methods. On Day 4, mice were treated intranasally with saline (VEH) or LPS (1 µg). Following 24 hour LPS challenge mice were sacrificed and (A) thymus weight was recorded. BAL was also performed and (B) total cell and (C) macrophages numbers were determined by differential cell counting analysis. δ Two-way ANOVA (Sal versus Dex, *p<0.05*). # One-way ANOVA (versus VEH, *p<0.05*).

Intraperitoneal administration of Dex (20 µg per day) over four days induced involution of the thymus, a positive control observation for *in vivo* steroid activity, resulting in a 58% decrease in thymus weight when compared with saline-treated mice (Figure 5A). CS and/or LPS did not significantly alter thymus weight and Dex-induced thymic involution was not altered by either stimulus. Sub-chronic CS exposure caused a significant increase in total BALF cell numbers (vehicle; 1±0.19 vs. CS; 3.6±0.18×10^5^ cells, *p<0.05*) that was completely refractory to Dex treatment (Figure 5B). LPS challenge increased total BALF numbers (10.2±1.1×10^5^ cells) in a manner that was reduced by Dex (49%). CS exposure prior to LPS reduced the efficacy of Dex, where only a 21% reduction in total cell numbers was observed. BALF macrophages were only increased in CS exposed mice (∼2-fold) that was not significantly altered by LPS or Dex treatment (Figure 5C).

### Neutrophil activation is associated with GC refractory MMP-9 release *in vivo*

**Figure 6:**
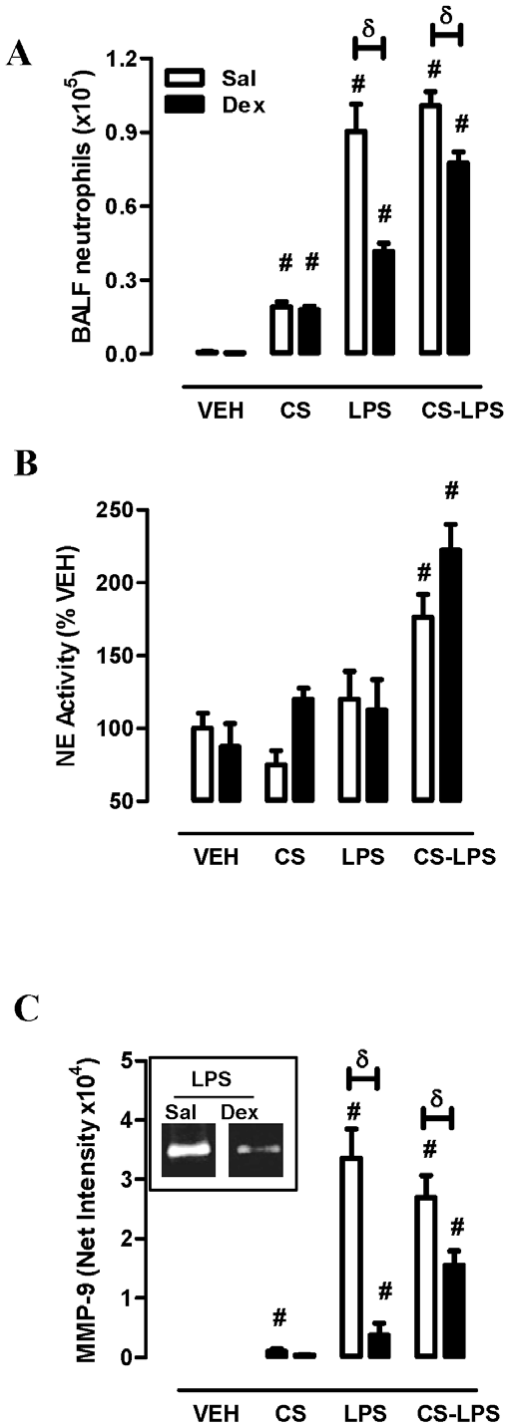
Differential MMP-9 and NE activity and altered GS responses in vivo. (A) BALF neutrophil numbers were determined from treatment groups as detailed in Figure 5 legend. BALF was retained and (B) NE activity and (C) MMP-9 expression (Insert: representative zymography results) was determined as detailed in methods (C) δ Two-way ANOVA (Sal versus Dex, *p<0.05*). # One-way ANOVA (versus VEH, *p<0.05*). The data was pooled from 2 separate experiments (n = 5–10 mice per treatment group).

CS exposure increased BAL neutrophil numbers (vehicle; 0.08±0.04 vs. CS; 1.9±0.21×10^5^, *p<0.05*) in a Dex insensitive manner (Figure 6A). LPS markedly increased BAL neutrophil numbers (9.022±1.1×10^5^ neutrophils/mL) and Dex suppressed this response by 54%. The combination of LPS and CS did not significantly alter the LPS neutrophilic response but did reduce the efficacy of Dex to 23%. NE activity in BALF was not significantly altered by CS, Dex or LPS treatment (Figure 6B). The combination of CS and LPS significantly increased NE activity (vehicle; 100±15.6 vs. CS-LPS; 176±15.6%, *p<0.05*). The response was further increased in the presence of Dex (222±17.7%). MMP-9 expression in BALF was modestly increased in CS exposed mice and insensitive to Dex (Figure 6C). There was a large increase in MMP-9 expression in LPS-challenged mice (vehicle; 30.9±10.7 vs. LPS; 33571±4930, *p<0.05*) and Dex suppressed this response by 89%. CS exposure prior to LPS reduced the efficacy of Dex, where a 42% reduction in MMP-9 was observed.

## Discussion

The close association between MMP-9 and BALF neutrophils in our COPD cohort identify this cell type as a major source of MMP-9. Increasing MMP-9 and NE with disease severity in the presence of increasing ICS dosage is consistent with enhanced neutrophil activation and degranulation. Our *in vitro* data demonstrates that fMLP stimulated blood neutrophils release granules containing MMP-9 and NE in a GC resistant manner. Furthermore, degranulation of NE and MMP-9 share similar signalling pathways (PI3K dependence), but diverge in their requirement for MAPK signalling, where only MMP-9 was reduced by Erk and p38 pathway inhibition. Our findings are consistent with previous observations that also identified MAPK (Erk1/2 and p38) [19] signalling as necessary for MMP-9 degranulation. The release of MMP-9 by neutrophils can also occur in response to other inflammatory mediators such as CXCL8 [20], TNFα [21] and endotoxin [22]. Unlike denovo MMP-9 production by other cell types such as macrophages, this response occurs rapidly and is released independently of TIMP1, as neutrophils do not produce this anti-proteinase [10]. MMP-9 is formed in the later stages of neutrophil maturation and this proteinase contributes to neutrophil extravasation and stem cell mobilisation [23], [24] via the degradation of basement membrane collagens, whereas NE is primarily responsible for pathogen killing (reviewed in [25]).

Primary azurophilic granules containing NE normally undergo limited exocytosis and are traditionally associated with the intracellular killing of microorganisms in the phagolysosome, as mice deficient in NE are more susceptible to gram negative bacteria [26]. The majority of NE is expressed on the neutrophil surface during activation, with less than 5% being released into the extracellular milieu [27]. In COPD, free NE activity may be further increased by necrotic neutrophils. *Haemophilus Influenzae* is a common gram negative pathogen in COPD that chronically colonises the airways and directly causes neutrophil necrosis and release of azurophilic granular content [28]. *In vivo*, only CS-primed neutrophils release free NE activity in response to LPS challenge in a manner that was completely refractory to GCs. The hierarchy of granular release is only partially understood and involves intracellular calcium changes that can differentially regulate degranulation, where azurophilic granule release is dependent on calcium transient, in contrast to CXCL8-mediated gelatinase exocytosis [29]. Calcium changes regulate soluble-*N*-ethylmaleimidesensitive-factor accessory-protein receptors (SNAREs), a system responsible for fusion of granules with the plasma membrane [30] and the density of SNAREs may regulate exocytosis potential. Components of CS such as nicotine have been shown to increase intracellular calcium levels in neutrophils [31], however it is not known whether this response further primes azurophilic exocytosis or whether the expression or function of SNAREs are altered with increasing COPD severity.

*In vivo*, MMP-9 release was maximal in response to LPS challenge in a manner that was effectively blocked by GCs. This occurred in the absence of neutrophil activation as determined by measuring concurrent NE release. The absence of neutrophil activation suggests that alternative cellular sources are producing MMP-9 in response to LPS alone, such as alveolar macrophages. Alveolar macrophages increase production of MMP-9 in response to LPS stimulation in a GC sensitive manner [32], which is consistent with our *in vivo* data. In COPD, epigenetic deregulation in COPD macrophages is associated with oxidant dependant loss of HDAC2 expression and subsequent loss of GC mediated suppression of inflammatory genes [33]. In addition, our data support neutrophil activation of as an alternative mechanism for GC refractoryMMP-9 release. We found that CS priming and LPS challenge promoted neutrophil activation and this was associated with a reduction in GC efficacy to block MMP-9 *in vivo*. This data suggests that there is a transition from macrophage to neutrophil mediated release of MMP-9 in the presence of GCs and is consistent with our *in vitro* findings where neutrophil derived MMP-9 was completely resistant to GCs. Reduced Dex actions may also initiate a positive feedback inflammatory loop, as matrix breakdown products further augment neutrophil release of MMP-9 [11].

Elevated NE levels may also directly contribute to ongoing inflammation. NE activates TLR-4 signalling to promote CXCL8 expression in bronchial epithelial cells via activation of NFκB [16], [34]. Whether NE directly engages TLR-4 or modifies a required surface co-receptor or adaptor molecule has yet to be elucidated. In addition, NE was identified at the apex of a signalling cascade that can promote CXCL8 release via EGFR signalling in bronchial epithelial cells [35]. This process involves the surface shedding of EGFR ligands that initiate receptor transactivation and subsequent activation of p38 and NFκB. EGFR transactivation is also implicated in other pathological processes in COPD. NE mediated activation of this pathway promotes airway mucin production [36]. Furthermore, we have previously shown that EGFR transactivation augments inflammatory responses initiated by rhinovirus infection of bronchial epithelial cells [37].

In summary, we found that both NE and MMP-9 activity was elevated in severe COPD and was closely related to neutrophilic inflammation. *In vivo*, neutrophil activation was associated with GC refractory release of MMP-9. *In vitro*, neutrophils rapidly release both proteinases in a GC resistant manner that was dependent on PI3K signalling. Maximal release of MMP-9, but not NE was also dependent on Erk and p38 signalling. Neutrophil activation may represent a fundamental mechanism of GC resistance in COPD, as NE is at the apex of inflammatory and mucus pathways and its release is completely resistant to GCs. By identifying the mechanisms for neutrophil derived NE and MMP-9 release in chronic inflammatory conditions and their lack of regulation by currently used anti-inflammatory agents such as GCs, better therapeutic strategies can be designed to combat deregulated inflammation in COPD.

## Methods

### Ethics Statement

This research was approved by the Hunter New England Human Research Ethics Committee of the Hunter New England Area Health Service (HNEAH). All participants gave informed written consent prior to their inclusion in this study. The animal experiments were approved by the Animal Ethics Committee (AEC) of the University of Melbourne and conducted in compliance with the National Health and Medical Research Council (NHMRC) of Australia.

### Subjects

Fiberoptic bronchoscopy was performed on subjects with stable COPD (*n* = 29; GOLD II (*n* = 9), GOLD III (*n* = 9) and GOLD IV (*n* = 11). Patient characteristics are summarized in Table 1. The percentage use and daily dosage of ICS at time of sampling is summarized in Table 1. Warmed saline (2×60 mL) was instilled into the airways to enable recovery of BAL cells and BAL fluid (BALF). BAL cells were collected by centrifugation (400× *g* for 5 min) and cytospot slides were prepared and stained for differential cell analysis based on morphology. Cell free BALF was archived at −80°C for future analysis.

### Proteinase Assays

Measurement of NE activity in BALF and supernatant was determined using the specific NE substrate methoxysuccinyl-alanyl-alanyl-prolyn-valylpapnitroanalide (MEOSAAPVNA, Sigma; St Louis, MO). Neat BALF (5 µL) was diluted to 200 µL NE Buffer containing final concentrations of 0.2 mM MEOSAAPVNA, 50 mM Tris-HCl (pH 7.5), 5 mM CaCl_2_, 1 mM ZnCl_2_ and 0.01% sodium azide. Absorbance at 405 nm was measured using microplate reader (MultiScan Ascent, Thermo).

Zymography was used to quantify gelatinase activity in BALF, which identified major gelatinase bands observed at ∼90 kDa corresponding to MMP-9 as previously published [38]. Briefly, gelatin substrate (2 mg/mL, LabChem Inc, Pittsburgh, PA) was incorporated into SDS polyacrylamide mini-gels (10%) before casting. BALF (10 µL) was run into gels at a constant voltage (200 V) under non-reducing conditions. Gels were washed twice in Triton X-100 solution (2.5%) for 15 minutes and incubated overnight at 37°C in zymography buffer (50 mM Tris-HCl (pH 7.5), 5 mM CaCl_2_, 1 mM ZnCl_2_ and 0.01% sodium azide). The gels were then stained with Coomassie Brilliant Blue R-250 (Sigma St Louis, MO) for 45 minutes and extensively destained. After destaining, zones of enzyme activity appeared clear against the background of Coomassie Blue stain.

Total MMP-9 levels (pro and active forms) were assessed using quantitative ELISA (DuoSet ELISA, RnD Systems) in accordance with the manufacturer's instructions. Measurement of MMP-9 activity in BALF and was determined using fluorescein labeled DQ gelatin substrate (Life Technologies), which is only cleaved by the active form of the enzyme. Neat BALF (40 µL) was diluted to 200 µL Buffer containing final concentrations of 3 µg fluorescein labeled DQ gelatin substrate, 50 mM Tris-HCl (pH 7.5), 5 mM CaCl_2_, 1 mM ZnCl_2_ and 0.01% sodium azide. Fluorescence intensity (relative fluorescence units, RFU) was measured using a fluoresecence microplate reader with standard fluorescein filters. Digested products from DQ Gelatin have absorption maxima of 495 nm and emission maxima of 515 nm.

### *In vitro* Analysis

All reagents were obtained from Sigma-Aldrich unless otherwise stated. Venous blood was collected upon receipt of written consent from healthy volunteers. Neutrophils were isolated by Ficoll-Paque density gradient centrifugation. The pellet enriched for erythrocytes and neutrophils was next mixed with an equal volume of 6% Dextran T500 in saline and allowed to sediment for 20 min. The neutrophil enriched upper layer was removed and contaminating erythrocytes were removed by hypotonic lysis. Cell viability was checked using Trypan blue exclusion method and was routinely found to be>95%. Neutrophil purity was confirmed by differential staining to be >95%. Neutrophils were resuspended to 2×10^6^ cells per mL in Krebs Buffer and incubated with 5 µg/mL Cytochalasin B for 15 min prior to stimulation. Isolated cells were also pretreated with Dex (100 nM), wortmannin (100 nM), U0126 (1 µM) or SB203580 (1 µM) at 37C for 15 min prior to fMLP stimulation (100 nM, 37C for 20 min). At the end of the incubation, cell free supernatant was retained by centrifugation at 1000 g for 5 min and archived at −80C prior to NE and MMP-9 analysis.

### *In vivo* Analysis

Specific pathogen-free male Balb/c mice aged 7 weeks and weighing ∼20 g were obtained from the Animal Resource Centre Pty. Ltd. (Perth, Australia). The animals were housed at 20°C on a 12-h day/night cycle in sterile micro-isolators and fed a standard sterile diet. Mice were treated with Dex (1 mg/kg, i.p.) for 2 hours prior to CS exposure. For the sub-chronic CS exposure protocols, mice were placed in an 18 liter perspex chamber in a class II biosafety cabinet as previously described [39] with some minor modifications. Briefly, mice were exposed to whole body sidestream CS generated from one cigarette (2R4F, University of Kentucky, Lexington, KY) for 15 min and allowed to recover for 2 hours.The levels of carboxyhemoglobin in the blood of Balb/c mice exposed to CS using this model have previously been measured and found to be comparable to that observed in smokers [39]. This process was repeated 2 times for a total of 3 cigarettes per day over four days. On the fourth day, the final CS exposure was replaced by treating the mice with LPS. For LPS challenge, mice were anesthetised by methoxyflurane (Medical Developments International Ltd, Springvale, Australia) inhalation and treated intranasally with 1 µg LPS diluted in 50 µL PBS. Saline was used as a control for Dex and LPS. Following 24 hours, thymus weight was also recorded as a quantitative measure of GC activity. BAL was also performed via tracheotomy (SP30 Duran polyethylene tubing) proximal to the larynx and total/differential BAL cell counts were performed as previously described [40]. Cell free BALF was archived at −80°C prior to analysis of MMP-9 and NE activity.

### Statistical analysis

Normally distributed data were expressed as mean ±S.E. For the clinical samples Mann Whitney U test was used to analyze the data. Spearman correlation was used to assess the relationship between secreted proteinases and neutrophil numbers in BALF and the relationship between MMP-9 and NE. For the animal studies, significance between groups was tested using either one-way or two-way analysis of variance (ANOVA) followed by Dunnetts comparison test where p≤0.05 was considered significant.
